# Neuroinflammation in Alzheimer’s disease: glial crosstalk, pathological modulation, and therapeutic implications

**DOI:** 10.3389/fimmu.2026.1783786

**Published:** 2026-05-29

**Authors:** Enhao Li, Peng Zhang, Wei Feng

**Affiliations:** 1 Department of Psychiatry, Chongqing University Three Gorges Hospital, Chongqing University, Chongqing, China; 2 Department of Geriatrics, Chongqing University Three Gorges Hospital, Chongqing University, Chongqing, China; 3 Department of Neurology, Chongqing University Three Gorges Hospital, Chongqing University, Chongqing, China

**Keywords:** Alzheimer’s disease, astrocytes, evidence hierarchy, glial crosstalk, microglia, neuroinflammation, therapeutic implications

## Abstract

Alzheimer’s disease (AD) remains a major therapeutic challenge despite the availability of amyloid-targeting disease-modifying therapies for selected patients with early symptomatic disease. These therapies have shown that disease modification is possible, but their benefits are modest and constrained by amyloid confirmation, safety monitoring, infusion delivery, access, and eligibility requirements. Neuroinflammation is increasingly viewed as a context-dependent modifying process that interacts with β-amyloid (Aβ), tau pathology, metabolic stress, and vascular dysfunction, rather than as an unequivocally established primary initiating driver. This review provides a selective, glia-centered synthesis of AD neuroinflammation focused on microglia, astrocytes, and their reciprocal crosstalk. We examine how microglial and astrocytic responses can support Aβ handling, plaque containment, tissue homeostasis, and synaptic protection, while chronic or poorly resolved glial signaling can amplify cytokine and complement responses, metabolic and oxidative stress, and neuronal vulnerability. Representative signaling nodes, including NF-κB, AMPK/mTOR, and PI3K/Akt, are discussed as organizing mechanisms linking inflammatory transcription, proteostatic stress, glial metabolism, and neuronal injury, rather than as equivalently validated therapeutic targets. Therapeutic implications are interpreted across three evidence tiers: approved anti-amyloid antibodies with indirect inflammatory relevance, clinically tested anti-inflammatory or immunomodulatory strategies that have not established disease-modifying efficacy, and experimental precision approaches aimed at glial-state modulation. Overall, the translational challenge is not broad suppression of neuroinflammation, but stage-specific identification and modulation of maladaptive glial states while preserving protective microglial and astrocytic functions.

## Graphical Abstract

**Figure:**
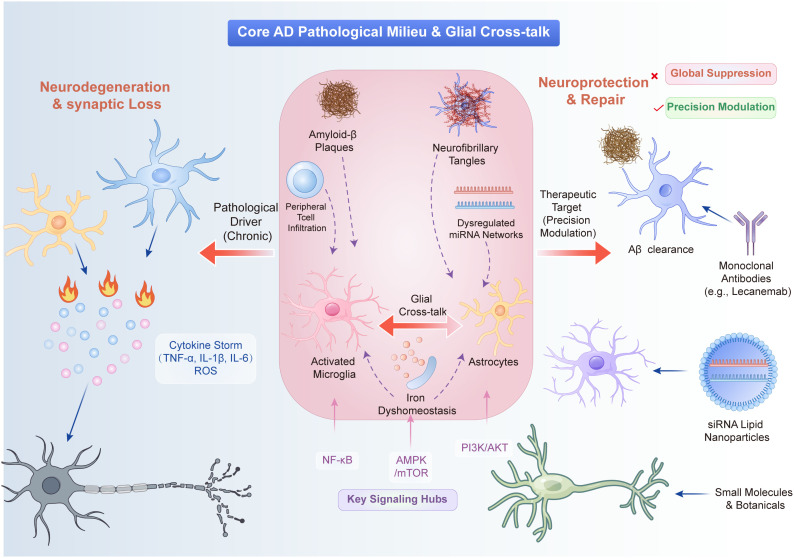
.

## 1 Introduction

### 1.1 Current therapeutic context and unmet needs in AD

Alzheimer’s disease (AD) is the leading cause of dementia and a growing public-health challenge as population aging increases the number of affected individuals worldwide (1). Clinically, AD is characterized by progressive decline in memory and other cognitive domains, accompanied over time by impairment in behavior, daily function, and independence (2).

The therapeutic landscape has changed, but remains constrained. Cholinesterase inhibitors and N-methyl-D-aspartate receptor antagonists provide symptomatic benefit but do not substantially modify the underlying neurodegenerative process (3). Amyloid-targeting monoclonal antibodies have introduced disease-modifying options for selected patients with early symptomatic AD (4, 5). In phase 3 trials, lecanemab and donanemab slowed clinical decline compared with placebo, and both have received U.S. regulatory approval for treatment initiation in patients with early symptomatic disease, including mild cognitive impairment or mild dementia due to AD (5). However, their benefits are modest and their use is constrained by amyloid-related imaging abnormalities, infusion and monitoring requirements, eligibility restrictions, cost, and access (6). These limitations sustain the need to define additional biological processes that modify disease progression and may guide complementary therapeutic development.

### 1.2 Aβ, tau, and neuroinflammation as a modifying process in AD pathogenesis

The defining neuropathological features of AD are extracellular β-amyloid (Aβ) plaques and intraneuronal neurofibrillary tangles composed of abnormally phosphorylated tau (7, 8). Although Aβ and tau remain central biological anchors for diagnosis, staging, and therapeutic development, AD pathogenesis is not adequately captured by a simple linear sequence from amyloid deposition to tau-mediated neurodegeneration (9, 10). Instead, AD is better understood as a multifactorial process in which protein aggregation, synaptic dysfunction, metabolic and vascular stress, genetic risk, and glial responses interact over time (10, 11).

Within this framework, neuroinflammation is best positioned as an important contributing and modifying process rather than as an unequivocally established primary initiating driver (10). In the central nervous system, AD-related inflammatory responses are organized mainly by microglia and astrocytes, which can support Aβ handling, plaque containment, tissue repair, and homeostatic regulation under certain conditions (12, 13). Conversely, when glial activation becomes chronic, spatially dysregulated, or poorly resolved, microglia–astrocyte signaling may amplify cytokine and complement responses, synaptic injury, metabolic stress, blood–brain barrier dysfunction, and neuronal vulnerability (14, 15). The pathogenic significance of neuroinflammation is therefore context-dependent: glial responses may be protective, compensatory, or maladaptive according to disease stage, local Aβ and tau burden, genetic background, and the surrounding metabolic and vascular environment (16–18).

### 1.3 Scope and focus of this review

Consistent with this context-dependent framing, this review does not attempt to catalogue all immune-related mechanisms, biomarkers, or putative anti-inflammatory interventions implicated in AD. Instead, it provides a selective, glia-centered synthesis of AD neuroinflammation, focusing on microglia–astrocyte crosstalk, representative signaling nodes that connect inflammation with proteostatic and metabolic stress, and therapeutic approaches that are interpretable according to evidence level and translational maturity. This structure is intended to distinguish mechanistic plausibility from clinical validation and to emphasize a central therapeutic problem: how to modulate maladaptive glial states without suppressing protective or compensatory glial functions. The overall glia-centered framework linking Aβ/tau pathology, microglia–astrocyte crosstalk, neuronal vulnerability, and therapeutic modulation is summarized in the **Graphical Abstract**.

## 2 Microglia and astrocytes as principal effector cells in AD neuroinflammation

AD-associated neuroinflammation is best organized around resident glial cells, particularly microglia and astrocytes, because these cells sense Aβ- and tau-associated stress, remodel plaque-adjacent niches, and coordinate immune, metabolic, and reparative responses (10, 15). In this section, cytokines, chemokines, complement factors, and oxidative or nitrosative mediators are treated as signals that shape glial state and intercellular communication, rather than as independent catalogues of inflammatory outputs. This framing keeps the section focused on cellular organization: how microglia and astrocytes respond to AD pathology, how their states are modified, and when these responses shift from adaptive containment toward injury amplification.

### 2.1 Microglia: plaque sensing, chemotaxis, and heterogeneous activation

Microglia are the resident immune cells of the CNS and maintain tissue surveillance under homeostatic conditions (10). In AD, microglial responses become plaque-associated and state-dependent, involving chemotaxis toward Aβ deposits, phagocytic handling of pathological material, barrier-like plaque containment, lipid-metabolic remodeling, and inflammatory signaling (13, 19). Aβ can activate microglial danger-sensing pathways such as TLR2-dependent signaling, but the broad release of IL-1β, TNF-α, IL-6, and oxidative mediators should be interpreted as one output of altered microglial state rather than as the main organizing theme of this subsection (20).

Chemokine signaling helps connect plaque localization with microglial or mononuclear phagocyte recruitment (21, 22). In AD mouse models, CCR2 deficiency impaired microglial or mononuclear phagocyte accumulation and worsened amyloid-related pathology, supporting the view that recruitment can contribute to plaque surveillance and clearance in some settings, even though excessive or persistent recruitment may amplify local inflammation (23, 24). Thus, microglial migration should be discussed as a context-dependent spatial response rather than as a uniformly detrimental inflammatory event.

Microglial activation in AD is also heterogeneous. The historical M1/M2 terminology is useful only as a simplified shorthand, because single-cell studies show that disease-related microglia occupy multiple and dynamic states rather than fixed binary phenotypes (25). A well-known example is the disease-associated microglia (DAM) program in amyloid mouse models, characterized by reduced homeostatic gene expression and increased expression of genes related to lipid metabolism, phagocytosis, and AD risk, including Trem2 and APOE (26). Human AD microglia show partial overlap with murine DAM-like signatures but also display human-specific and region-specific programs, underscoring that mouse-derived glial categories should not be transferred uncritically to human AD (27, 28).

**Figure 1:**
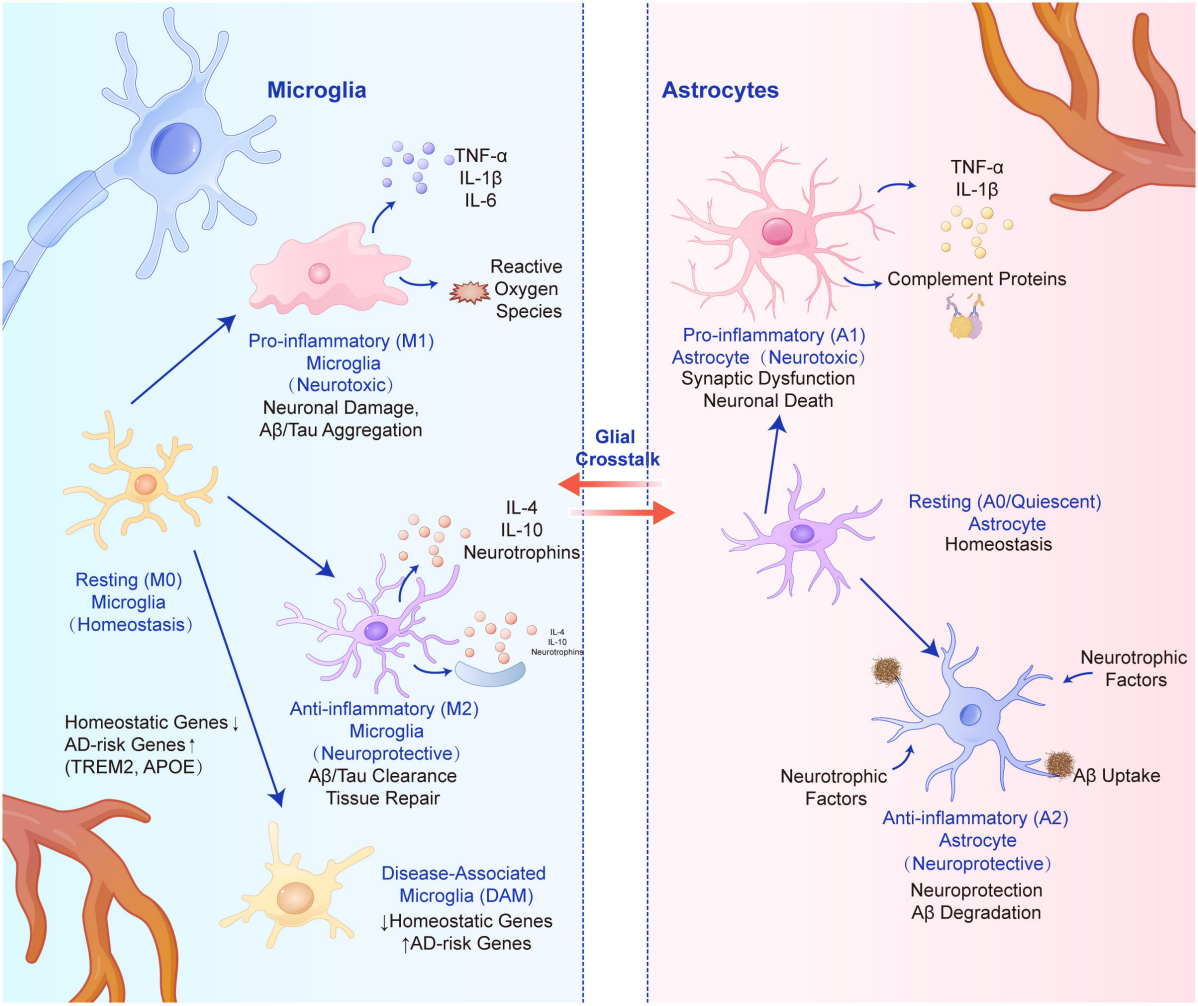
Context-dependent glial responses in AD neuroinflammation. The M1/M2 and A1/A2 labels are shown only as simplified historical descriptors and should not be interpreted as discrete or fixed *in vivo* states. Microglial and astrocytic responses in AD exist along heterogeneous and dynamic continua shaped by disease stage, local Aβ/tau burden, genetic background, and the surrounding metabolic and vascular environment.

The APOE/TREM2 axis is therefore treated here as a genetic–glial modifier of microglial plaque responses. TREM2-related programs support microglial lipid handling, metabolic fitness, and responses to amyloid pathology, whereas ApoE can facilitate the microglial response to plaques and influence innate immune signaling (29, 30). ApoE-containing lipoproteins can also interact with TREM2 and facilitate microglial uptake of Aβ, linking lipid signaling with plaque-associated phagocytic function (31). The central message is that microglia in AD should be defined by where they act, which state program they engage, and whether their response supports containment or amplifies injury. The context-dependent spectrum of microglial and astrocytic responses in AD neuroinflammation is summarized in **Figure 1**.

### 2.2 Astrocytes: reactive states, complement signaling, and homeostatic failure

Astrocytes support synaptic and neurotransmitter homeostasis, metabolic coupling, blood–brain barrier integrity, and neurovascular regulation, but only the AD-relevant aspects of these functions are developed here (32, 33). In AD, astrocytes undergo reactive remodeling that includes morphological, molecular, metabolic, and inflammatory changes rather than a single stereotyped activation pattern (34). Because fixed A1/A2 categories oversimplify astrocyte biology, reactive astrocytes should be described as heterogeneous states shaped by disease stage, brain region, plaque proximity, vascular context, and local glial signaling (35, 36).

Microglia-to-astrocyte signaling provides a concrete mechanism for this state remodeling. Activated microglia-derived IL-1α, TNF-α, and C1q can induce neurotoxic reactive astrocyte states, linking microglial activation with astrocytic loss of homeostatic support and injury-associated inflammatory programs (14). However, this pathway should not be taken to mean that all reactive astrocytes are harmful; astrocytes can also participate in Aβ clearance, plaque-related tissue responses, and metabolic support (12, 37).

Complement signaling illustrates how astrocytic reactivity can become part of reciprocal glial amplification. Astrocyte-derived complement C3 can signal through microglial C3aR and modulate amyloid pathology in AD mouse models, whereas C3 activation in human AD brain and mouse models has been associated with neurodegeneration (38, 39). Spatial transcriptomic evidence further supports the view that microglia and astrocytes form localized interacting responses around amyloid plaque niches, rather than functioning as isolated inflammatory cell types (15).

ApoE also links astrocyte biology to microglial plaque responses because astrocytes are a major CNS source of ApoE, and APOE genotype shapes lipid handling, Aβ clearance, inflammatory responsiveness, and glial communication (40, 41). ApoE therefore links astrocyte biology with microglial plaque responses through lipid transport, Aβ handling, and inflammatory modulation.

### 2.3 Modifiers of glial responses: inflammatory mediators and peripheral immune inputs

Cytokines and chemokines are retained here only as modifiers of glial state. In AD models, Aβ burden is associated with elevations of TNF-α, IL-6, and IL-1-family cytokines, and Aβ-stimulated microglia can produce inflammatory mediators through danger-sensing pathways (20, 42). Such mediators can also feed back onto amyloid-related biology; for example, IL-1β, TNF-α, and IFN-γ have been reported to stimulate γ-secretase-mediated APP cleavage in experimental systems (43). These observations support a feed-forward link between protein pathology and glial inflammatory tone, but they do not imply that nonspecific cytokine suppression is clinically validated in AD.

Oxidative and nitrosative mediators are reduced here to one stress-modifying point: nitric oxide-related pathways and Aβ nitration have been linked experimentally to amyloid deposition or aggregation, but these mechanisms should not be expanded into a stand-alone ROS/RNS mini-review in a principal effector-cell section (44, 45). Peripheral immune activity may also reshape glial responses; studies have reported clonal CD8+ T-cell responses in AD cerebrospinal fluid and T-cell infiltration linked to neurodegeneration in tauopathy models (46, 47). These observations justify acknowledging peripheral immune input without shifting the focus away from microglia and astrocytes.

Taken together, microglia and astrocytes are the central cellular organizers of AD neuroinflammation. Cytokines, chemokines, complement factors, oxidative or nitrosative mediators, ApoE/TREM2-related genetic influences, and peripheral immune inputs are best viewed as modifiers of glial state rather than as parallel subjects requiring independent review treatment. This cellular framing helps distinguish adaptive responses, such as plaque surveillance, Aβ handling, and tissue support, from maladaptive responses, such as persistent complement activation, inflammatory amplification, and loss of homeostatic glial function.

## 3 Microglia–astrocyte crosstalk as a context-dependent modifier of AD pathology

Microglia and astrocytes form localized communication networks in AD rather than acting as isolated inflammatory cell populations (15, 48). Spatial transcriptomic and single-cell studies identify plaque-associated niches in which microglial and astrocytic states are coordinated around Aβ deposition and tissue injury (15, 36). These interactions can constrain pathology, as illustrated by astrocytic IL-3-dependent programming of microglial plaque responses, but they can also amplify injury through complement-dependent synapse loss and inflammatory glial loops (49, 50). Their pathological significance depends on disease stage, local Aβ and tau burden, genetic background, and the surrounding vascular and metabolic environment (51).

### 3.1 Representative molecular routes of microglia–astrocyte crosstalk

Activated microglia can induce injury-associated astrocytic states through IL-1α, TNF-α, and C1q signaling (14). This mechanism is relevant to AD because inflammatory and complement-related astrocyte programs are observed in human AD and around amyloid pathology, although astrocytic responses remain regionally and stage dependent rather than uniformly neurotoxic (51).

Astrocytic complement C3 provides a reciprocal route from astrocytes back to microglia (38). In AD mouse models, astrocyte–microglia complement activation modulates amyloid pathology, and NF-κB-driven astroglial C3 release has been linked to neuronal structural and functional impairment (38, 52). Complement also connects glial crosstalk with synaptic vulnerability, because complement and microglia mediate early synapse loss in AD mouse models (50).

Lipid-related genetic signaling provides another major route of crosstalk. Astrocyte-derived ApoE shapes Aβ pathology and DAM-like microglial responses in experimental systems, while lipidated ApoE can bind TREM2 and facilitate microglial Aβ uptake (31, 53). TREM2-dependent programs maintain microglial metabolic fitness and support plaque-associated microglial phenotypes, linking astrocytic lipid signaling with microglial responses to amyloid pathology (29, 54).

Astrocyte-derived IL-3 illustrates the adaptive side of astrocyte-to-microglia communication. In AD models, astrocytic IL-3 programmed microglia toward an Aβ-responsive phenotype that enhanced plaque-related immune functions and limited AD-like pathology (49). Together with cytokine, complement, and lipid-related routes, IL-3 highlights that glial crosstalk can redirect microglial responses rather than simply intensify inflammation.

Across these routes, three recurring motifs are most relevant: cytokine/complement signaling that can amplify injury, lipid/genetic signaling that tunes plaque-associated microglial states, and astrocyte-derived cues that can restrain or redirect microglial responses. The same pathway may have different consequences across disease stages and local niches; therefore, glial crosstalk is better evaluated by cell state, location, and timing than by a fixed protective/toxic label.

### 3.2 Metabolic, redox, and iron-related stress as glial-state modifiers

Iron and redox stress modify microglia–astrocyte communication by altering the metabolic and inflammatory environment of glial cells. In APP/PS1 mice, inflammatory microglia have been described as glycolytic and iron-retentive, and microglial iron accumulation has been linked to altered metabolism and impaired function (55, 56). Iron can potentiate Aβ-induced microglial IL-1β secretion, connecting iron handling with cytokine amplification (57). Astrocytic iron regulation provides a complementary route: astrocyte-derived hepcidin attenuated brain iron deposition, oxidative stress, and neuronal loss in APP/PS1 mice (58).

Ferroptosis-related biology provides a mechanistic language for iron-dependent lipid peroxidation, but its AD relevance remains mainly experimental. Ferroptosis is defined by iron-dependent cell death and lipid peroxide accumulation, with GPX4 functioning as a key antioxidant-defense node (59, 60). AD studies have linked iron dyshomeostasis, lipid peroxidation, ferroportin loss, and ferroptosis-like injury to cognitive and pathological phenotypes, supporting their role as stress modifiers within the glial microenvironment (61, 62). These findings justify brief consideration of iron/redox stress, but they do not establish iron chelation, ferroptosis inhibition, or antioxidant therapy as clinically validated AD treatments.

### 3.3 Selected regulatory layers of glial crosstalk

MicroRNAs can regulate glial inflammatory tone, and miR-146-related studies in Aβ- or lipopolysaccharide-associated models illustrate post-transcriptional control of inflammatory signaling; however, this evidence remains too preliminary to support a separate therapeutic discussion (63, 64).

Calcineurin/nuclear factor of activated T cells (CN/NFAT) signaling represents a calcium-dependent transcriptional layer of glial regulation. NFATc2 can modulate microglial activation in AβPP/PS1 mice, indicating that calcium-dependent transcriptional signaling shapes microglial inflammatory phenotype (65). Astrocytic CN/NFAT signaling is also relevant to glial dysfunction, because astrocyte-targeted inhibition or modulation of this pathway reduced glial activation, network abnormalities, or AD-related functional changes in Aβ-bearing mouse models (66, 67).

PD-L1/PD-1 signaling provides an immune-checkpoint-like layer within astrocyte–microglia communication. In APP/PS1 mice, astrocytic PD-L1 stimulation of microglial PD-1 suppressed neuroinflammation and AD-like pathology (68). Reports on PD-1 pathway manipulation in AD and tauopathy models have been mixed, supporting cautious interpretation rather than assuming that checkpoint blockade or stimulation will be uniformly beneficial (69, 70).

Overall, microglia–astrocyte crosstalk in AD is best understood through a limited set of recurring motifs: cytokine/complement signaling, lipid-related genetic modulation, metabolic and redox stress, and selected regulatory checkpoints. These mechanisms are not equivalent therapeutic targets; they represent interacting layers that shape whether glial responses support plaque containment and homeostasis or amplify inflammatory injury and neuronal vulnerability.

## 4 Selected signaling nodes linking glial activation, metabolic stress, and neuronal vulnerability

NF-κB, AMPK/mTOR, and PI3K/Akt are useful organizing nodes because each links glial state changes to a different dimension of AD pathology. NF-κB connects plaque- and tau-associated stress with inflammatory transcription in microglia and astrocytes, including mediator programs that affect complement release and tau propagation (71, 72). AMPK/mTOR links energy sensing with autophagy, Aβ handling, and inflammasome regulation in glial cells (73, 74). PI3K/Akt connects neuronal stress responses and tau-related kinase signaling with microglial migration and Aβ phagocytosis (75, 76). These pathways are not equivalent therapeutic targets; their value is that they organize mechanistic connections among glial activation, proteostatic stress, metabolism, and neuronal vulnerability.

### 4.1 NF-κB signaling as a glial inflammatory transcriptional coordinator

NF-κB is best understood in AD neuroinflammation as a glial transcriptional coordinator rather than as a generic inflammatory marker. In a tauopathy mouse model, microglial NF-κB activation promoted tau spreading and toxicity, whereas pathway inactivation reduced tau seeding and modified tau-associated microglial disease states (71). In astrocytes, NF-κB activation has been linked to AD-relevant inflammatory remodeling, and astroglial NF-κB can induce complement C3 release that disrupts neuronal morphology and function (52, 72).

This pathway is particularly useful as an organizing node because it connects upstream danger sensing with downstream cytokine, chemokine, and complement outputs. Selective disruption of TLR2–MyD88 signaling attenuated inflammation and AD-like pathology in experimental models, illustrating how innate immune inputs can converge on NF-κB-associated inflammatory programs (77). Astrocytic NF-κB/C3 signaling also connects intracellular inflammatory transcription with complement-mediated astrocyte–microglia crosstalk (52). Chronic engagement of this axis may therefore maintain feed-forward glial reactivity and increase neuronal vulnerability, without implying that NF-κB is a primary initiating driver of AD.

**Figure 2:**
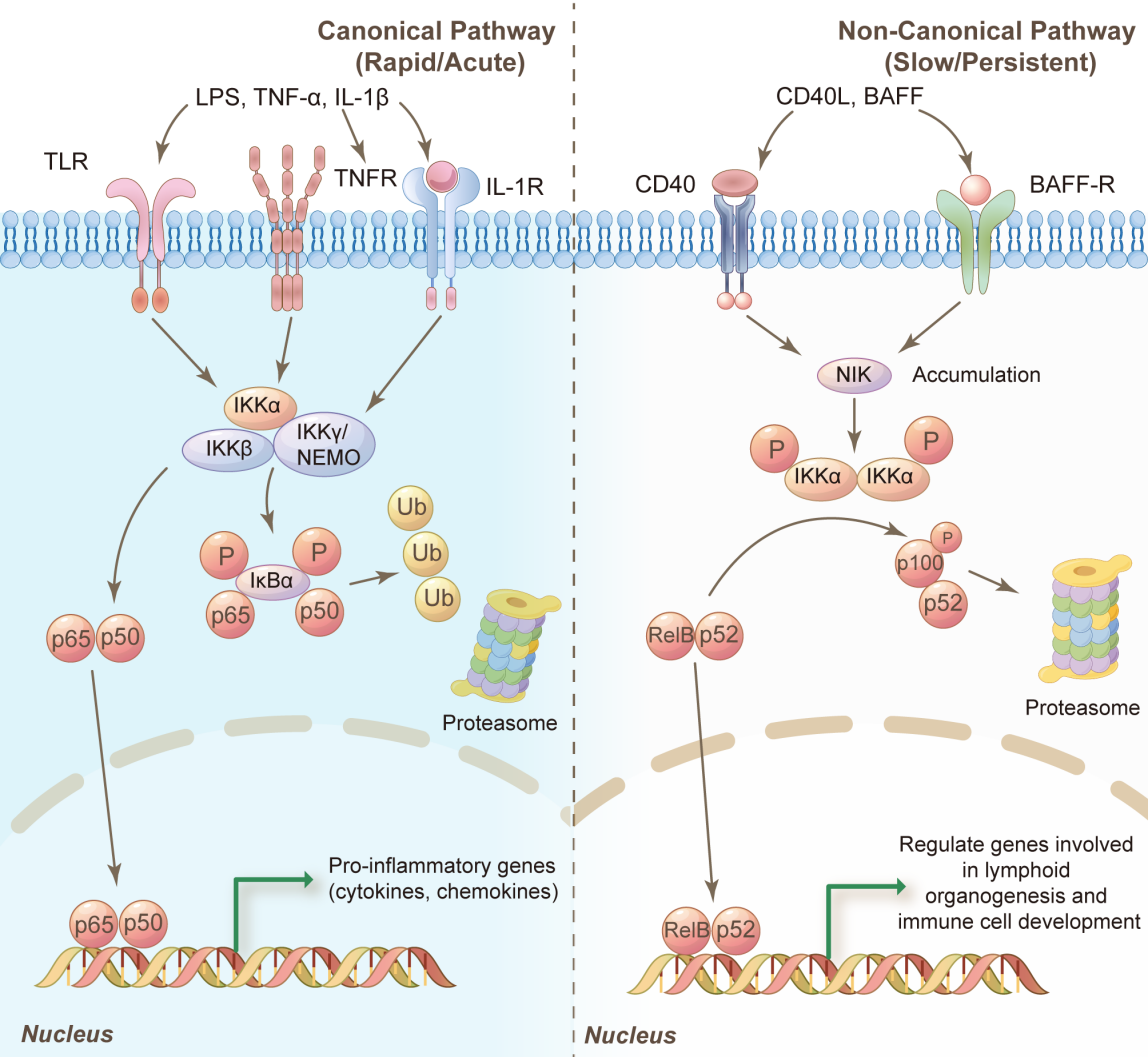
Canonical and non-canonical NF-κB signaling as inflammatory transcriptional pathways. The canonical pathway can be activated by inflammatory stimuli such as TLR, TNFR, and IL-1R signaling, leading to p65/p50-mediated transcription of inflammatory mediator genes. The non-canonical pathway involves NIK-dependent processing of p100 to p52 and RelB/p52-mediated transcriptional regulation. In this review, NF-κB is discussed as a glial inflammatory transcriptional coordinator relevant to AD neuroinflammation, rather than as a clinically validated stand-alone therapeutic target.

Mechanistic modulation of NF-κB-related pathways can clarify AD-relevant inflammatory biology, but current evidence does not establish NF-κB-directed intervention as a clinically validated AD therapy. The main interpretive value of NF-κB in this review is that it explains how persistent glial inflammatory transcription can connect Aβ- or tau-associated stress with complement activation, mediator release, and neuronal injury. The canonical and non-canonical NF-κB pathways linking inflammatory receptor activation to transcriptional outputs are illustrated in **Figure 2**.

### 4.2 AMPK/mTOR signaling as a metabolic-autophagic regulatory axis

AMPK and mTOR coordinate cellular energy sensing and autophagic initiation. AMPK can promote autophagy through phosphorylation of ULK1 under energy stress, whereas mTORC1 suppresses autophagy through regulation of the ULK1–ATG13–FIP200 complex under nutrient-replete conditions (73, 78). In AD-related glial biology, this axis is most informative when it connects autophagic flux with Aβ handling and inflammatory tone.

Microglial autophagy provides the clearest glia-centered example. Autophagy in microglia degrades extracellular Aβ fibrils and regulates Aβ-induced NLRP3 inflammasome activation (74). Aβ can also activate microglial NLRP3 inflammasomes through Syk-dependent AMPK inhibition and metabolic or mitochondrial dysfunction (79). More recent work indicates that autophagy enables microglia to engage amyloid plaques and prevents microglial senescence in AD mouse models (80). These findings make AMPK/mTOR an integrative node linking metabolic state, plaque engagement, autophagic capacity, and inflammatory signaling.

Timing and cell-type specificity limit therapeutic interpretation. Long-term rapamycin treatment reduced Aβ42 levels and prevented cognitive deficits in PDAPP mice, while in 3xTg-AD mice rapamycin improved pathology and cognition when given before plaque and tangle formation but not after established pathology (81, 82). In APP/PSEN1 astrocytes, MTORC1 inhibition enhanced autophagy-mediated Aβ clearance, whereas AMPK activation did not; in neurons, AMPK activation likewise did not directly enhance autophagy-dependent Aβ clearance (83, 84). These observations argue against simple therapeutic formulations such as uniformly activating AMPK or inhibiting mTOR across all AD contexts.

### 4.3 PI3K/Akt signaling as a context-dependent stress-response pathway

PI3K/Akt is included as a stress-response node because it occupies different positions in neurons and microglia. Intraneuronal Aβ42 can downregulate Akt survival signaling and blunt stress responses, whereas altered Akt–GSK3β signaling has been linked to tau phosphorylation (75, 85). These neuronal data support the relevance of PI3K/Akt to cell survival and tau-associated stress, but they do not support a uniformly protective interpretation.

In microglia, PI3K/Akt-related signaling can regulate Aβ handling and motility. Cathepsin B was reported to modulate microglial migration and Aβ phagocytosis through PI3K/Akt signaling, placing this pathway within plaque-associated immune function (76). This example links PI3K/Akt to glial behavior more directly than broad claims about neuronal survival alone.

The pathway’s context dependence remains essential. Experimental reduction of Akt activation protected neuronal cultures against synthetic Aβ peptides, indicating that stronger Akt activity is not automatically beneficial (86). PI3K/Akt should therefore be described as a cell- and stage-dependent stress-response node connecting Aβ toxicity, tau-related kinase signaling, microglial phagocytosis, and neuronal vulnerability, not as a validated disease-modifying target in AD.

Together, these three signaling nodes clarify how glial activation can become coupled to proteostatic and metabolic stress. NF-κB organizes inflammatory transcription, AMPK/mTOR organizes metabolic-autophagic handling of protein stress, and PI3K/Akt bridges microglial plaque-related functions with neuronal survival and tau-associated signaling. Their shared value is interpretive rather than therapeutic: they help explain how glial responses may shift from adaptive stress handling toward persistent inflammatory amplification and neuronal vulnerability.

## 5 Therapeutic implications across three evidence tiers

Therapeutic implications of AD neuroinflammation are clearest when organized by evidentiary strength and translational maturity. The first tier includes approved anti-amyloid disease-modifying therapies, whose inflammatory relevance is indirect and mediated mainly through amyloid lowering and plaque-niche remodeling (5). The second tier includes clinically tested anti-inflammatory or immunomodulatory strategies, where trial outcomes have been negative, inconclusive, or insufficient for validation (87). The third tier includes precision approaches designed to modify glial states, which remain proof-of-concept rather than established therapeutic options (88). This hierarchy separates clinical efficacy from biological plausibility and helps avoid treating all anti-inflammatory mechanisms as equivalent therapeutic opportunities.

### 5.1 Approved disease-modifying therapies with indirect inflammatory relevance

Anti-amyloid monoclonal antibodies currently provide the strongest clinical evidence for disease modification in selected patients with early symptomatic AD. In CLARITY AD, lecanemab reduced amyloid markers and produced moderately less clinical decline at 18 months than placebo; FDA traditional approval specifies use in patients with confirmed amyloid pathology and treatment initiation at the mild cognitive impairment or mild dementia stage. In TRAILBLAZER-ALZ 2, donanemab significantly slowed clinical progression at 76 weeks in participants with early symptomatic AD and amyloid/tau pathology; FDA approval similarly specifies treatment initiation in patients with mild cognitive impairment or mild dementia (5). Their use remains constrained by eligibility requirements, infusion delivery, amyloid confirmation, ARIA risk, and MRI monitoring.

The inflammatory relevance of these antibodies is secondary to amyloid removal. Studies of Aβ immunization and lecanemab suggest that microglial effector functions contribute to amyloid clearance and plaque-niche remodeling (89, 90). However, the approved therapeutic target remains Aβ pathology rather than inflammatory signaling itself, so these agents do not validate broad anti-inflammatory therapy as a disease-modifying strategy (4, 5).

### 5.2 Clinically tested anti-inflammatory strategies that have not yet succeeded

Clinical experience with broad anti-inflammatory suppression has been unfavorable. In the Alzheimer’s Disease Anti-inflammatory Prevention Trial, naproxen and celecoxib did not improve cognitive function, with weak evidence for a detrimental cognitive effect of naproxen (91). A randomized placebo-controlled prednisone trial found no difference in cognitive decline between prednisone and placebo, with behavioral decline observed in the prednisone group (87). These findings argue against nonspecific inflammatory suppression, particularly when disease stage, glial state, target engagement, and compensatory immune functions are not clearly defined.

More targeted immunomodulatory strategies have not closed this validation gap. A randomized phase 2 trial of subcutaneous etanercept demonstrated tolerability in AD dementia but did not establish definitive disease-modifying efficacy (92). In mild AD, the p38α inhibitor neflamapimod did not improve episodic memory over 24 weeks, although cerebrospinal fluid biomarker changes suggested biological activity (93). GV-971 occupies a separate category: a 36-week phase 3 trial in China reported cognitive benefit in mild-to-moderate AD, but the proposed gut microbiota-associated anti-inflammatory mechanism remains debated and the global confirmatory phase 3 trial was terminated early (94).

Together, these clinical examples support a narrower translational message. The problem is not simply that inflammatory pathways are irrelevant, but that broad or weakly stratified interventions have not shown reliable clinical benefit. Future anti-inflammatory strategies will need to identify maladaptive glial states, demonstrate target engagement, select appropriate disease stages, and avoid impairing protective microglial or astrocytic functions.

### 5.3 Experimental precision-targeting approaches that remain proof-of-concept

Microglia-targeted RNA modulation illustrates a future precision-targeting tier. Ralvenius et al. developed microglia-tropic lipid nanoparticles for localized intracisternal delivery of anti-PU.1 siRNA and reported reduction of PU.1 levels and neuroinflammatory programs in human stem cell-derived microglia-like cells and animal models of neuroinflammation (88). This approach is mechanistically relevant because PU.1/SPI1 regulates microglial gene expression and AD-associated inflammatory responses (95, 96).

This platform remains early translational. Major unresolved issues include delivery route, durability of knockdown, safety, cell-type specificity, target engagement in human AD brain, and whether modulation of microglial transcriptional states improves clinically meaningful outcomes (88). Microglia-targeted siRNA is therefore best positioned as an experimental tool for precision modulation of glial inflammatory programs, not as an established pharmacotherapeutic strategy for AD.

Taken together, the therapeutic literature supports an evidence-tiered interpretation of AD neuroinflammation. Approved anti-amyloid antibodies demonstrate disease modification in selected early symptomatic AD, but their relationship to inflammation is indirect. Direct anti-inflammatory or immunomodulatory strategies have not yet produced a validated disease-modifying therapy for AD, and experimental glial-state targeting remains proof-of-concept. The translational challenge is therefore to move from broad suppression of neuroinflammation toward stage-specific modulation of maladaptive glial states while preserving protective glial functions.

**Table 1** summarizes selected therapeutic approaches with direct or indirect relevance to AD neuroinflammation. These approaches are grouped according to clinical maturity, relationship to neuroinflammation, evidentiary strength, and major limitations; the table is not intended as a comprehensive catalogue of anti-inflammatory or immunomodulatory interventions in AD.

**Table 1:** Selected therapeutic strategies with direct or indirect relevance to AD neuroinflammation: clinical status and evidentiary strength.

Evidence tier/clinical status	Representative therapy or approach	Relationship to neuroinflammation	Balanced summary	References
Approved amyloid-targeting disease-modifying therapy	Lecanemab	Indirectly influences neuroinflammation through amyloid lowering	Modestly slows clinical decline in carefully selected patients with early symptomatic AD, but requires monitoring because of ARIA and other adverse events.	(4, 97)
	Donanemab	Indirectly influences neuroinflammation through amyloid lowering	Modestly slows clinical decline in selected patients with early symptomatic AD, with important safety and monitoring considerations including ARIA.	(5)
Investigational strategies with a more direct anti-inflammatory rationale	NSAIDs/COX inhibition	Suppression of prostaglandin signaling, glial activation, and inflammatory mediator production	Mechanistic rationale and preclinical support exist, but disease-modifying efficacy in AD has not been established.	(98–104)
	Glucocorticoids	Broad immunosuppressive and anti-inflammatory effects on glia and inflammatory mediators	Dose-dependent effects have been reported in preclinical models; current clinical evidence in AD is limited, and prednisone did not demonstrate benefit.	(87, 105, 106)
Mechanistically relevant but clinically unproven	Anti-TNF biologics	TNF pathway modulation	Mechanistic rationale and preclinical data exist, but convincing clinical efficacy data in AD are lacking.	(107, 108)
Regionally approved but debated	Sodium oligomannate (GV-971)	Proposed effects on gut microbiota, inflammatory mediators, and Aβ-related pathways	Reported cognitive benefit in China, but broader acceptance remains limited and its mechanism and therapeutic significance remain controversial.	(94, 109, 110)
Experimental proof-of-concept	Microglia-targeted siRNA nanoparticles (MG-LNP/anti-PU.1)	Direct modulation of microglial inflammatory programs	Represents an early proof-of-concept in AD mice, but substantial translational barriers remain.	(88)
Mechanistically interesting but unproven	p38α MAPK inhibition	Modulation of inflammatory kinase signaling	Preclinical findings are mixed, and the currently cited evidence does not establish clinical efficacy in AD.	(93, 111–114)

## 6 Conclusions and perspectives

Neuroinflammation in AD is best understood as a context-dependent modifying process rather than as a single primary initiating driver or a process suitable for global suppression. Microglia and astrocytes can support Aβ handling, plaque containment, tissue homeostasis, and synaptic protection, but chronic or poorly resolved microglia–astrocyte signaling can also amplify cytokine, complement, metabolic, and oxidative stress responses that increase neuronal vulnerability. This functional ambivalence argues against classifying glial activation as simply protective or harmful.

A glia-centered framework provides a more coherent interpretation than a catalogue of isolated mediators or pathways. Spatially organized microglia–astrocyte interactions around amyloid plaque niches, complement-dependent synaptic injury, lipid-related genetic modulation, inflammatory transcription, and metabolic-autophagic stress are best viewed as interacting glial-state modifiers rather than equivalent therapeutic targets. Their pathological significance depends on cell state, local Aβ and tau burden, disease stage, genetic background, and the surrounding vascular and metabolic environment.

The therapeutic literature reinforces the gap between mechanistic plausibility and clinical validation. Lecanemab and donanemab have shown that disease modification is possible in selected patients with early symptomatic AD, but their inflammatory relevance is indirect and mediated mainly through amyloid lowering and plaque-niche remodeling. By contrast, interventions directed more explicitly at inflammatory pathways have not yet produced a validated anti-inflammatory disease-modifying therapy for AD, as illustrated by negative or inconclusive clinical experience with NSAIDs, prednisone, and pathway-focused approaches such as p38α inhibition.

Future development should therefore move from broad anti-inflammatory logic toward stage-specific modulation of maladaptive glial states. The key translational questions are which glial programs are protective, which become harmful, when this transition occurs, and how these states can be measured and modified in patients. Progress will require biomarkers that distinguish protective from maladaptive glial responses, trial designs that incorporate disease stage and molecular target engagement, and therapeutic strategies that preserve homeostatic glial functions while limiting inflammatory amplification.
